# Genome-wide identification and expression analyses of genes involved in raffinose accumulation in sesame

**DOI:** 10.1038/s41598-018-22585-2

**Published:** 2018-03-12

**Authors:** Jun You, Yanyan Wang, Yujuan Zhang, Komivi Dossa, Donghua Li, Rong Zhou, Linhai Wang, Xiurong Zhang

**Affiliations:** 1 Key Laboratory of Biology and Genetic Improvement of Oil Crops, Ministry of Agriculture, Oil Crops Research Institute, Chinese Academy of Agricultural Sciences, Wuhan, 430062 China; 2 Special Economic Crop Research Center of Shandon Academy of Agricultural Sciences, Shandong Cotton Research Center, Jinan, 250100 China; 3 Centre d’Etudes Régional pour l’Amélioration de l’Adaptation à la Sécheresse (CERAAS), BP 3320 Route de Khombole, Thiès, Senegal

## Abstract

Sesame (*Sesamum indicum* L.) is an important oilseed crop. However, multiple abiotic stresses severely affect sesame growth and production. Raffinose family oligosaccharides (RFOs), such as raffinose and stachyose, play an important role in desiccation tolerance of plants and developing seeds. In the present study, three types of key enzymes, galactinol synthase (GolS), raffinose synthase (RafS) and stachyose synthase (StaS), responsible for the biosynthesis of RFOs were identified at the genome-wide scale in sesame. A total of 7 *SiGolS* and 15 *SiRS* genes were identified in the sesame genome. Transcriptome analyses showed that *SiGolS* and *SiRS* genes exhibited distinct expression profiles in different tissues and seed developmental stages. Comparative expression analyses under various abiotic stresses indicated that most of *SiGolS* and *SiRS* genes were significantly regulated by drought, osmotic, salt, and waterlogging stresses, but slightly affected by cold stress. The up-regulation of several *SiGolS* and *SiRS* genes by multiple abiotic stresses suggested their active implication in sesame abiotic stress responses. Taken together, these results shed light on the RFOs-mediated abiotic stress resistance in sesame and provide a useful framework for improving abiotic stress resistance of sesame through genetic engineering.

## Introduction

Plant growth and productivity are greatly challenged by diverse environmental stresses, such as drought, waterlogging, or high-salinity, for their sessile nature. To cope with these unfavorable conditions, plants have evolved a range of physiological and biochemical responses by activating a large number of stress-responsive genes and synthesizing various functional proteins through an intricate network of signaling cascades^1,2^. At the cellular level, compatible solutes including sugars (such as oligosaccharides, trehalose, and sorbitol), amines (such as glycine betaine and polyamines), and amino acids (such as proline) accumulate under stress conditions and function as osmolytes or antioxidants that help plants to overcome harmful environmental conditions^3^.

Raffinose family oligosaccharides (RFOs), such as raffinose (Raf) and stachyose (Sta), are α-1, 6-galactosyl extensions of sucrose (Suc) and ubiquitous in plants^4^. RFOs (both Raf and Sta) predominantly accumulate in seeds, protecting the embryo against desiccation in seed maturation and thus play a critical role in prolonging seed longevity^5,6^. Accumulation of Raf is also found in vegetative tissues (leaves and roots) under abiotic stresses and function as an osmolyte, and/or an antioxidant to promote cell survival under adverse growing conditions^7–9^. Some evidence showed that RFOs may function as signals that mediate stress responses by activating the expression of downstream stress-responsive genes^10^. In addition, RFOs also participate in several important cellular biological processes, including transport and storage of carbon, and membrane trafficking^8,11,12^.

The first step in the RFOs biosynthesis starts with the formation of galactinol (Gol; 1-*O*-α-D-galactopyranosyl-*myo*-inositol), which is catalyzed by galactinol synthase (GolS, EC 2.4.1.123), using UDP-galactose and *myo*-inositol (Ino) as substrates^13^. Then, Raf and Sta are synthesized by stepwise addition of galactosyl units that are catalyzed by raffinose synthase (RafS, EC 2.4.1.82) and stachyose synthase (StaS, EC 2.4.1.67), respectively. RafS transfers a galactosyl unit from Gol to Suc and produces Raf^14^. Then, StaS further uses Gol as a galactosyl donor to Raf and yields Sta^15,16^. Both these two reactions are reversible^4^.

RFOs biosynthesis genes, especially *GolS* genes, have been well characterized in many plant species, including *Arabidopsis*^7,17^, tomato^18^, rice^19^, maize^20^ and poplar^21^. Most of the *GolS* genes were reported to be induced by stress stimuli and positively associated with abiotic stress resistance in plants. Among seven *GolS* genes (*AtGolS1-7*) identified in *Arabidopsis*, *AtGolS1* and *AtGolS2* were up-regulated by salt and drought stresses, while *AtGolS3* was up-regulated by cold stress^17^. *AtGolS1*- or *AtGolS2*-overexpressing transgenic *Arabidopsis* plants with increased endogenous Gol and Raf showed enhanced tolerance to drought, salt, chilling and oxidative stresses^7,17^. Recently, Selvaraj *et al*. reported that AtGolS2 was able to confer drought resistance and increase grain yield in two different rice genotypes under dry field conditions^22^. Two cDNAs encoding GolS from wheat (*TaGolS1* and *TaGolS2*) were transformed into rice. The transgenic plants constitutively overexpressing *TaGolS1* or *TaGolS2* accumulated significantly higher levels of Gol and Raf, and exhibited enhanced cold stress tolerance compared with the untransformed control plants^23^. Above results suggest that *GolS* genes are good targets to improve the abiotic stress tolerance of crop plants through molecular breeding and/or genetic engineering. Compared to GolSs, RafSs and StaSs are poorly characterized in plants. Six putative *RafS* genes (*AtRS1-6*) were identified in *Arabidopsis*. Biochemical and genetic analyses indicated that *AtRS5* was the only *RafS* gene responsible for Raf accumulation in leaves under abiotic stresses^14^. Another *RafS* gene, *AtRS4*, encodes a seed specific multifunctional enzyme with RafS and high affinity StaS activity^15^.

Sesame (*Sesamum indicum* L.), an ancient oil crop, is widely grown in tropical and subtropical areas. Sesame is known as an important source of oil with an excellent nutritional quality due to its high content of oil and protein in the seeds, and the balance in composition of oleic and linoleic acids^24,25^. As an important species from the order Lamiales, the release of the full genome sequence of sesame has provided the useful genomic platform for the genetic improvement of sesame^26^. Although sesame is considered to be a relatively drought-resistant crop, it is highly sensitive to drought stress during its vegetative stage and its yield potential is often limited by water shortage^27,28^. RFOs are widely known for their important roles in various abiotic stresses resistance and seed development. However, the accumulation of these oligosaccharides in sesame is largely unknown. In the present study, genes involved in RFOs accumulation, including genes encoding GolS, RafS and StaS, were identified at the genome-wide scale in sesame. Then, a detailed gene structure, phylogenetic analyses were performed. Furthermore, the expression profiles of these genes in different organs and abiotic stresses were assessed, which provided useful information for identification of several *SiGolSs* and *SiRSs* as candidate genes for further functional analysis and genetic improvement of sesame.

## Results

### Identification of raffinose biosynthesis pathway genes in sesame

Seven *GolS* genes were identified from the sesame genome database (Sinbase, http://ocri-genomics.org/Sinbase/index.html) by a BLAST search using the protein sequence of GolSs from *Arabidopsis*. As shown in Table 1, we named the obtained *GolS* sequences *SiGolS1* to *SiGolS7* according to their positions from the top to the bottom on the sesame linkage groups (LGs). Fifteen putative raffinose synthase genes were also identified in the sesame genome, and designated as *SiRS1* to *SiRS15*. All the identified SiGolSs and SiRSs were checked manually for the presence of Glyco_trans_8 Pfam (PF01501) and Raffinose synthase Pfam (PF05691), respectively. The detailed information of *SiGolS* and *SiRS* genes, including locus ID, linkage group distribution, the length of coding sequences, molecular weight (MW), and theoretical isoelectric point (pI) is listed in Table 1.

**Table 1:** Raffinose biosynthesis related genes in Sesame.

Gene name	Locus ID^a)^	Accession Number^b)^	Linkage Group	Start (bp)	End (bp)	Exon number	Protein length (aa)	MW* (kDa)	pI*	Duplications
**GolS**										
SiGolS1	SIN_1018104	XM_011072789	LG02	17749734	17751585	3	339	38.41	4.82	Segmental
SiGolS2	SIN_1016153	XM_011073345	LG03	204802	205977	4	290	33.96	4.98	Dispersed
SiGolS3	SIN_1012641	XM_011081210	LG06	237773	239341	4	340	38.71	5.79	Segmental
SiGolS4	SIN_1026653	XM_011088281	LG08	11164540	11166854	4	333	37.97	4.94	Segmental
SiGolS5	SIN_1022773	XM_011089209	LG08	16345511	16347799	3	336	37.70	4.96	Segmental
SiGolS6	SIN_1022774	XM_011089210	LG08	16353437	16354675	3	306	35.07	4.75	Tandem
SiGolS7	SIN_1025925	XM_011093877	LG10	13337551	13339272	3	322	36.92	5.44	Segmental
**RafS**										
SiRS1	SIN_1020329	XM_011073865	LG03	4924917	4928632	4	779	86.75	6.60	Dispersed
SiRS2	SIN_1020252	XM_011073696	LG03	5530701	5534982	13	884	97.39	6.15	Segmental
SiRS3	SIN_1020251	XM_011073698	LG03	5545742	5549293	14	761	83.93	5.27	Tandem
SiRS4	SIN_1006202	XM_011076928	LG04	940755	945069	12	768	84.26	5.61	Segmental
SiRS5	SIN_1013540	XM_011080080	LG05	5970786	5974158	5	865	94.85	6.07	Segmental
SiRS6	SIN_1023532	XM_020693952	LG05	13038353	13041972	12	768	84.35	5.79	Segmental
SiRS8	SIN_1007330	XM_011085808	LG07	6751484	6754372	4	765	84.68	5.79	Segmental
SiRS9	SIN_1007333	XM_011085817	LG07	6786084	6789045	4	755	83.69	5.97	Proximal
SiRS10	SIN_1026883	XM_011087990	LG08	9189292	9193596	14	792	87.42	5.50	Dispersed
SiRS11	SIN_1026329	NA	LG08	14107419	14108200	3	198	21.36	5.12	Segmental
SiRS12	SIN_1026328	XM_011088697	LG08	14110902	14114536	14	750	82.35	5.21	Tandem
SiRS13	SIN_1014290	XM_011097309	LG12	1710058	1713804	12	789	87.31	5.64	Dispersed
SiRS14	SIN_1007882	XM_011100134	LG15	130561	133205	4	784	86.59	5.50	Dispersed
**StaS**										
SiRS7	SIN_1012629	XM_011081231	LG06	315049	317941	4	862	95.64	6.07	Dispersed
SiRS15	SIN_1003607	XM_011102449	scaffold00109	425302	429067	4	847	94.96	5.83	NA

^a)^Locus ID was adopted from Sinbase (*Sesamum indicum* genome database, http://ocri-genomics.org/Sinbase/index.html).

^b)^Accession Number was adopted from NCBI (The National Center for Biotechnology Information, https://www.ncbi.nlm.nih.gov/).

*MW: Molecular Weight; pI: Isoelectric point; NA: not available.

**Figure 1:**
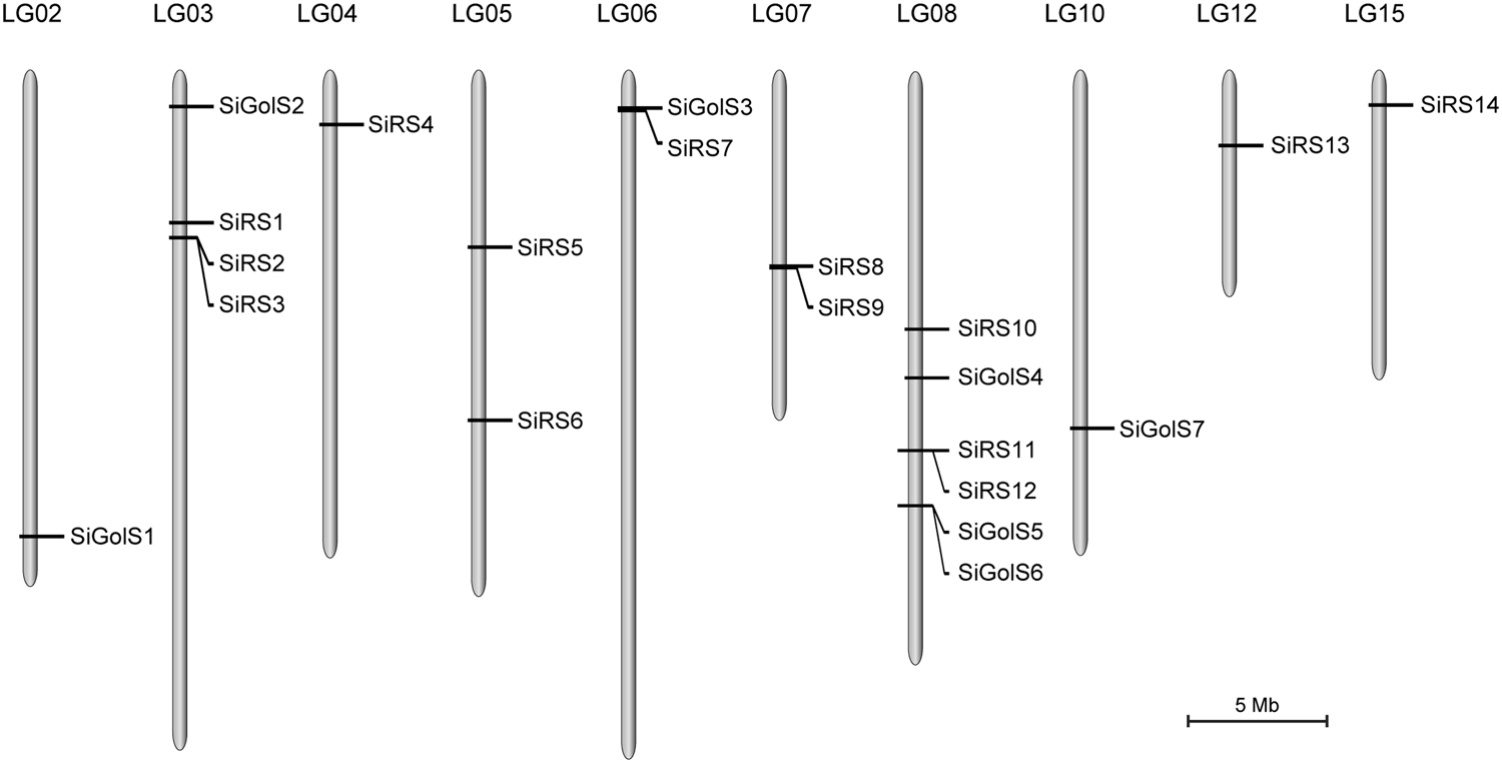
Linkage group distribution of *SiGolS* and *SiRS* genes in sesame. Totally 7 *SiGolS* and 15 *SiRS* genes were mapped to the 10 linkage groups (LGs) according to their positions in the sesame genome. The LG number was shown on the top of each LG. The scale bar indicated a LG distance of 5 Mb.

*SiGolS* and *SiRS* genes were mapped to the 16 sesame linkage groups based on the coordinates of Sinbase loci. As shown in Fig. 1, all the *SiGolS* and *SiRS* genes were unevenly distributed among 10 LGs out of the 16 LGs of the sesame genome, except for *SiRS15*, which was located on the unanchored scaffold. Sequencing analysis of the sesame genome revealed that the recent sesame whole genome duplication genomic regions covered approximately 50% of the current sesame genome assembly^26^. We further analyzed the segmental duplication events of *SiGolS* and *SiRS* genes. A total of 5 *SiGolSs* (*SiGolS1*, *3*, *4*, *5*, and *7*) and 6 *SiRSs* (*SiRS2*, *4*, *5*, *6*, *8*, and *11*) were detected as segmentally duplicated genes. As shown in Supplementary Fig. S1, these segmentally duplicated *SiGolS* and *SiRS* genes were located on duplicated segments on 8 LGs.

### Structural and phylogenetic analyses of SiGolSs

The predicted SiGolS proteins varied from 290 (SiGolS2) to 340 (SiGolS3) amino acids in length. Multiple sequence alignment of SiGolSs along with other reference GolS proteins from representative plant species, such as *Arabidopsis*, rice, poplar and soybean showed that all the GolS proteins have the glycosyl transferase 8 family domain (PF01501) (Supplementary Fig. S2). Except for SiGolS2, all of the reported GolS and SiGolS proteins contain a putative serine phosphorylation site at position 270 of the SiGolS1 protein. Except for SiGolS2 and SiGolS6, all of the SiGolSs contain the C-terminal hydrophobic pentapeptide (APSAA), a common feature of GolSs. Moreover, SiGolS5 shows a single substitution of proline to serine in APSAA pentapeptide (Supplementary Fig. S2).

**Figure 2:**
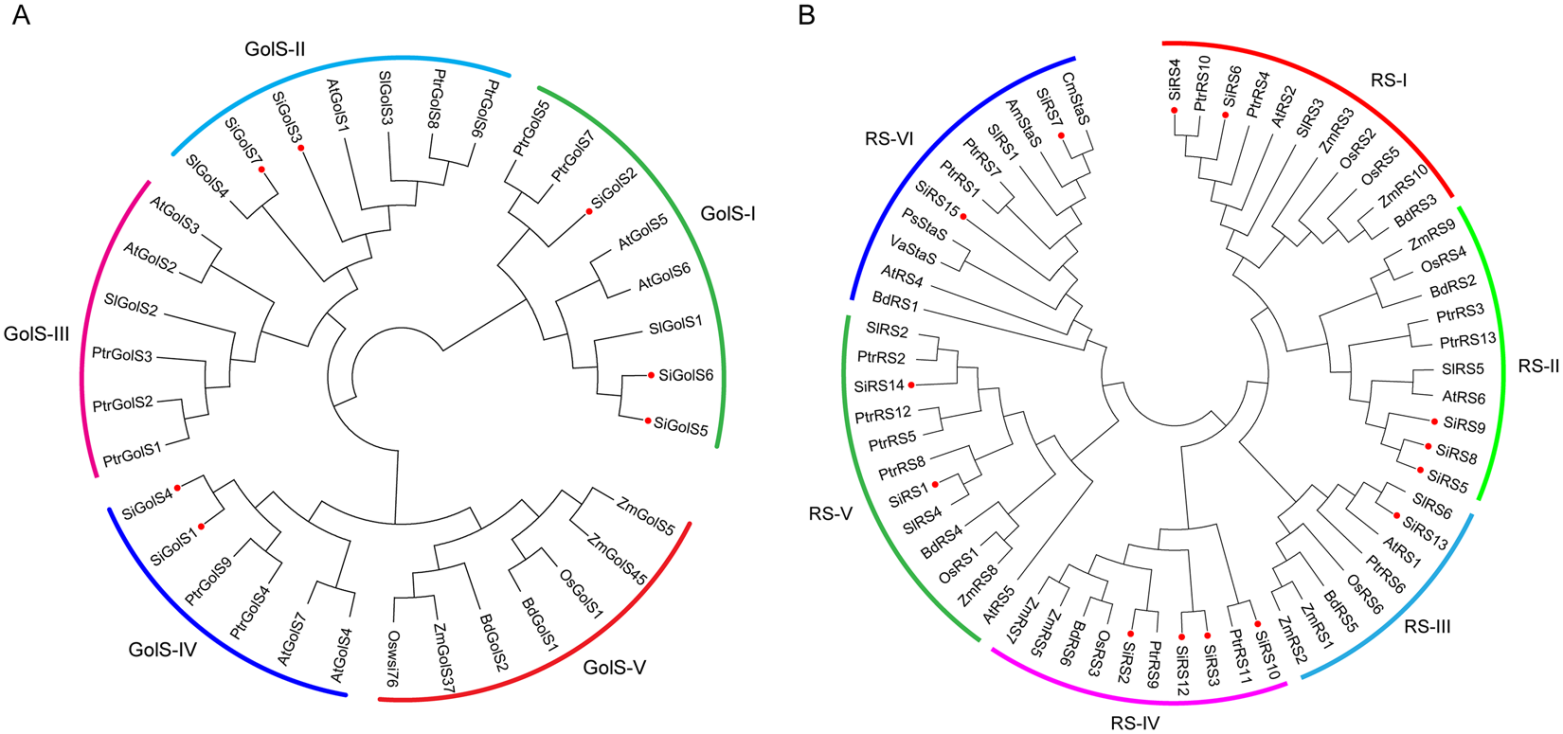
Phylogenetic analysis of GolSs and RSs from sesame and other species. (**A**) The deduced full-length amino acid sequences of 7 SiGolSs and 27 GolSs from 6 representative sequenced plant species (see Supplementary Table S2) were used for phylogenetic tree construction. (**B**) The deduced full-length amino acid sequences of 14 SiRSs, 44 RSs from six representative sequenced plant species (see Supplementary Table S3) and 4 referenece StaSs from *Vigna angularis* (VaStaS, CAB64363), *Pisum sativum* (PsStaS, CAC38094), *Cucumis melo* (CmStaS, XP_008451468) and *Alonsoa meridionalis* (AmStaS, CAD31704) were used for phylogenetic tree construction. The phylogenetic tree was constructed using MEGA 5.05 by the Neighbor-joining method with 1,000 bootstrap replicates. Members of GolS and RS proteins from sesame were denoted in red dots.

To investigate the evolutionary relationship of GolSs from sesame and other plant species, a Neighbor-joining tree was created based on the protein sequences of 34 GolSs from sesame, *Arabidopsis* (AtGolS1-7), tomato (SlGolS1-4), maize (ZmGolS1-3), poplar (PtrGolS1-9), rice (Oswsi76 and OsGolS1), and *Brachypodium distachyon* (BdGolS1 and BdGolS2). GolS proteins could be classified into 5 groups (GolS-I to GolS-V) according to the phylogenetic tree (Fig. 2A). SiGolS proteins were distributed in all groups, except GolS-III and GolS-V. Generally, SiGolSs have a closer relationship with SlGolSs as compared to AtGolSs, in accordance with the current understanding in their evolutionary history^26^. Notably, in the clade GolS-V, no GolS homologs were found from sesame, *Arabidopsis*, tomato and poplar (Fig. 2A), suggesting that group GolS-V is specific for monocot species.

To obtain further insight into the structural features of *SiGolS* genes, the exon/intron organization was analyzed by GSDS v2.0. As shown in Figure S3B, all *SiGolSs* have 3 or 4 exons. To better understand the evolution of SiGolSs, 10 conserved motifs were captured by MEME v4.11.0 (Supplementary Fig. S3C), and the details of the sequence logo of each motif were presented in Supplementary Fig. S4. Generally, SiGolSs in the same subfamilies showed similar motifs, indicating that the classification of SiGolS families was supported by motif analyses.

### Structural and phylogenetic analysis of SiRSs

The amino acid residues, molecular weight (MW) and theoretical isoelectric point (pI) of the 15 SiRS proteins were largely different, ranging from 198 aa/21.36 kDa (SiRS11) to 884 aa/97.39 kDa (SiRS2); 5.12 pI (SiRS11) to 6.60 pI (SiRS1) (Table 1). Among the *SiRS* genes, *SiRS11* only codes for approximately 25% of the length of a RafS or StaS protein (Table 1). Furthermore, the 198 amino acids of *SiRS11* correspond to partial Raffinose_synthase domain, and show only 48% identity with its closest relatives, SiRS12. Therefore, *SiRS11* and its putative product were not included in further analyses. Multiple sequence alignment of SiRSs along with reference RafS and StaS from other plant species, such as *Arabidopsis*, rice, *Vigna angularis* and *Cucumis melo* showed that all the SiRS protein sequences harbor the conserved domains of the Raffinose_synthase family (PF05691) (Supplementary Fig. S5). Although both RafS and StaS contain the same domain (Raffinose_synthase domain, PF05691), one sequence block of about 80 amino acids length present exclusively in StaS sequences is characteristic for StaS^15^. Based on the multiple sequence alignment, two SiRSs (SiRS7 and SiRS15), that containing the characteristic insertion of StaS, were identified as putative StaS encoding genes (Supplementary Fig. S5). Other thirteen SiRSs belong to the putative RafS enzyme family.

Phylogenetic analysis based on the full-length amino acid sequences of RSs from sesame and six other plants clearly distinguished RSs into 6 groups (RS-I to RS-VI) (Fig. 2B). SiRS proteins were distributed in all groups. Similar to SiGolSs, SiRSs have a closer relationship with RSs from tomato and poplar. Two putative StaSs in sesame (SiRS7 and SiRS15) were clustered with all other reference StaSs from *Arabidopsis* (AtRS4), *Vigna angularis* (VaStaS), *Pisum sativum* (PsStaS), *Cucumis melo* (CmStaS) and *Alonsoa meridionalis* (AmStaS) in group RS-VI^15,29,30^, indicating the group RS-VI might be specific for StaS enzyme family.

Exon-intron organization of the *SiRS* family was also investigated to reveal their gene structural diversity (Supplementary Fig. S6B). The numbers of intron of *SiRSs* varied from 3 to 14. In general, *SiRSs* clustered in the same group showed similar gene structure (Supplementary Fig. S6B). All *SiRS* genes in the group RS-II have 4 or 5 exons, while *SiRSs* in the group IV have 13 or 14 exons. Then, the MEME program was used to predict putative conserved motifs in SiRSs. A total of 20 putative motifs were detected (Supplementary Figs S6C and S7). As expected, SiRSs in the same groups have similar motif organization, indicating the link between evolutionary relationship and conserved motifs.

### Expression profiles of *SiGolS* and *SiRS* genes in different tissues

**Figure 3:**
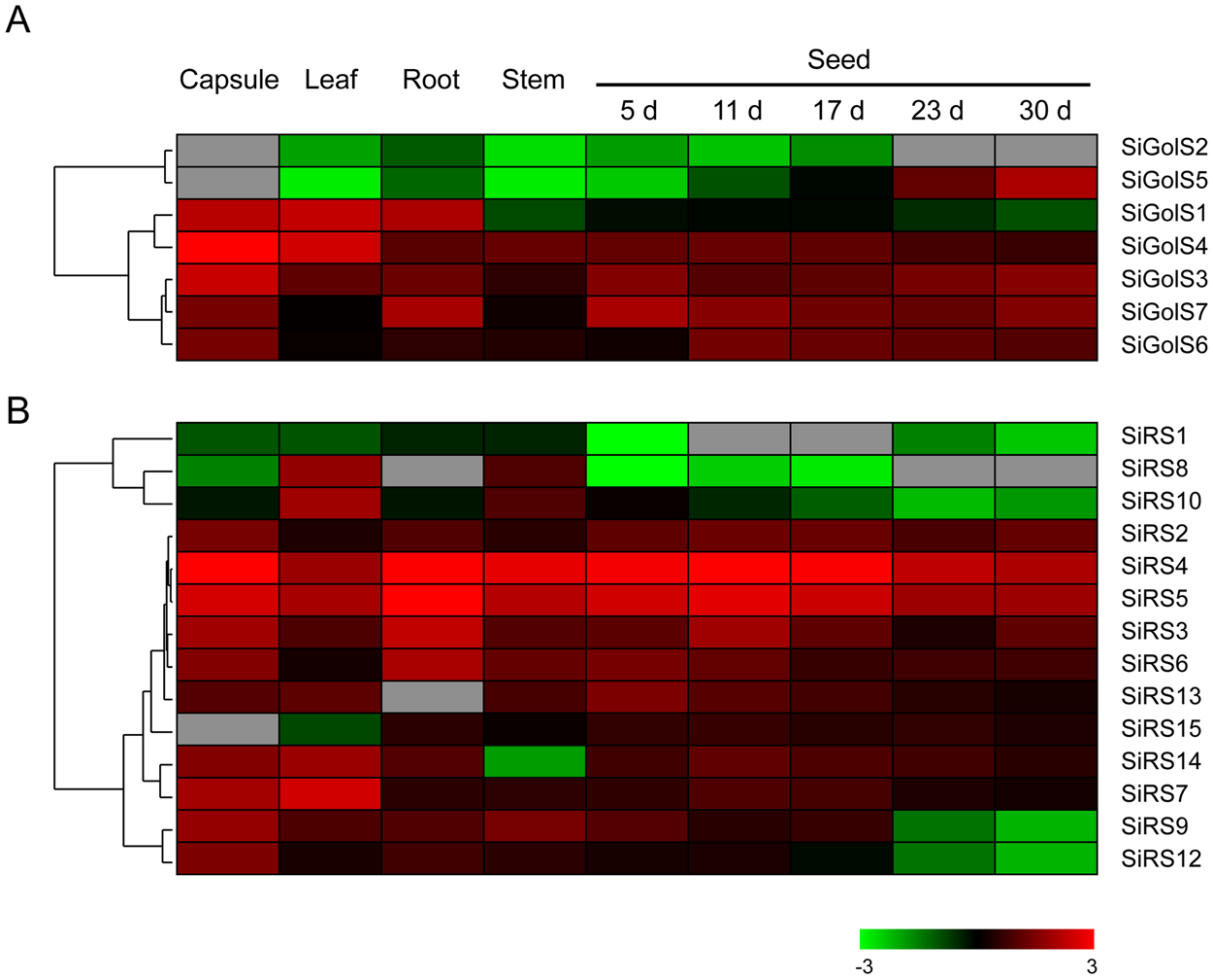
Expression profiles of the *SiGolS* and *SiRS* genes in different tissues. Transcriptome data downloaded from Sesame Functional Genomics Database (SesameFG, http://www.ncgr.ac.cn/SesameFG) was used to measure the expression level of *SiGolS* (**A**) and *SiRS* (**B**) genes in capsule, leaf, root, stem and seed at different stage of development. Heat maps were constructed by Cluster 3.0 based on the log_10_-transformed RPKM values for each gene. Gray box indicated the expression data is not available in this organ or time point. The color scale for expression values is shown.

To investigate the expression patterns of *SiGolS* and *SiRS* genes, their transcript levels in four tissue samples (capsule, leaf, root and stem) and seed samples at different developmental stages were retrieved from Sesame Functional Genomics Database (SesameFG, http://www.ncgr.ac.cn/SesameFG). Heatmaps were generated according to hierarchical clustering methods based on the RPKM values for each gene (Fig. 3). All *SiGolS* and *SiRS* genes displayed very diverse expression in all samples, except for *SiRS11*, which was not expressed across all tissues.

Among the 7 *SiGolS* genes, *SiGolS3* and *SiGolS4* displayed high expression, whereas, *SiGolS2* and *SiGolS5* showed relatively low expression in almost all tissues (Fig. 3A). *SiGolS1* displayed high expression levels in capsule, leaf and root, showed relatively low expression levels in stem and during the seed development. *SiGolS6* and *SiGolS7* exhibited high expression levels during the seed development and relatively low expression levels in leaf and stem (Fig. 3A).

Concerning the *SiRS* genes, all of them exhibited high expression levels in all tissues and developing seeds, except that *SiRS1*, 8 and 10 displayed relatively low expression levels during seed development (Fig. 3B). Especially, *SiRS4* and *SiRS5* were constitutively expressed at a relatively high level across all tissues. *SiRS8* and *SiRS10* exhibited specific high expression in leaf and stem. While, *SiRS14* and *SiRS15* exhibited specific low expression in stem and leaf, respectively. It is worth noting that over half of the *SiRS* genes displayed lower expression at the late stage of seed development compared to the early stage (Fig. 3B).

### Expression profiles of *SiGolS* and *SiRS* genes in response to abiotic stresses

**Figure 4:**
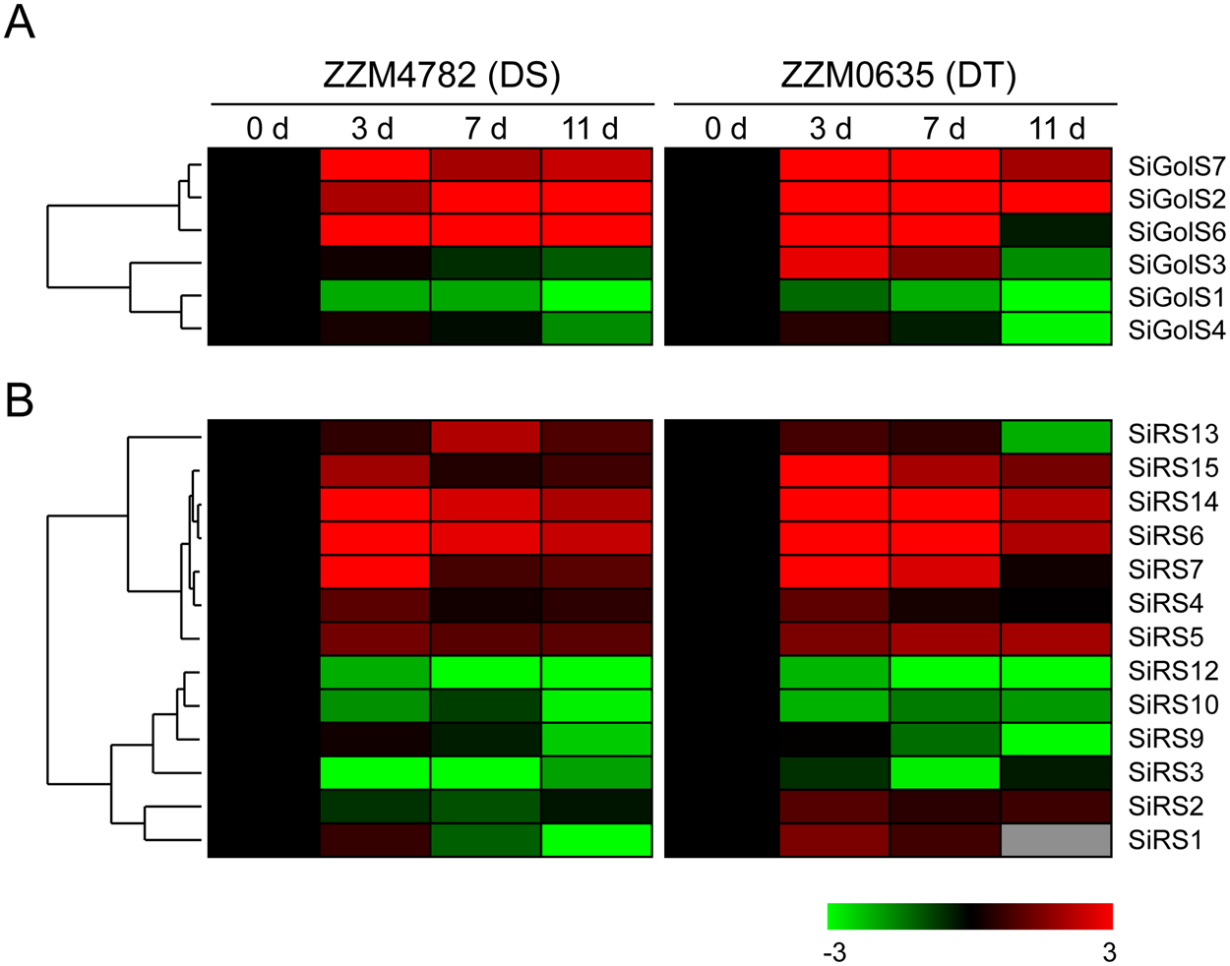
Expression profiles of *SiGolS* and *SiRS* genes under drought stress. The log_2_-transformed values (mean of three replicates) of the relative expression levels of the *SiGolS* (**A**) and *SiRS* (**B**) genes under drought stress in ZZM4782 (drought-sensitive, DS) and ZZM0635 (drought-tolerant, DT) varieties were used for creating the heatmap using Cluster 3.0. Gray box indicated the gene-expression change is not available at this time point. Changes in gene expression are shown in color as the scale.

**Figure 5:**
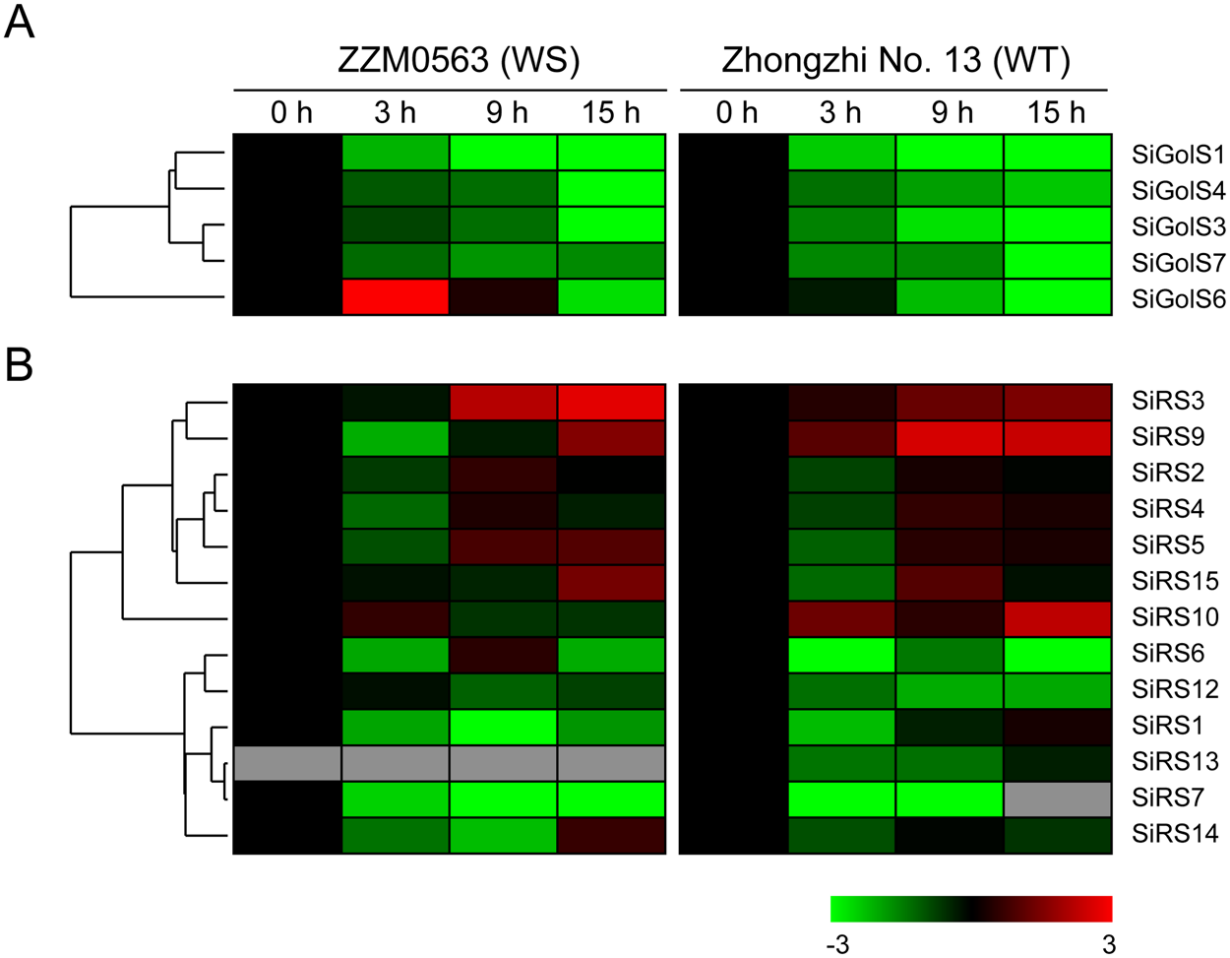
Expression profiles of *SiGolS* and *SiRS* genes under waterlogging stress. The log_2_-transformed values (mean of three replicates) of the relative expression levels of the *SiGolS* (**A**) and *SiRS* (**B**) genes under waterlogging stress in Zhongzhi No. 13 (waterlogging-tolerant, WT) and ZZM0563 (waterlogging-susceptible, WS) varieties were used for creating the heatmap using Cluster 3.0. Gray box indicated the gene-expression change is not available at this time point. Changes in gene expression are shown in color as the scale.

*GolS* and *RS* genes have been reported for their responsiveness to various abiotic stresses^14,17^. Thus, the expression patterns of these genes in response to drought and waterlogging stresses in the root of genotypes with contrasting tolerance levels were firstly revealed by two separate transcriptome analyses^31,32^. According to the transcriptome data, 6 *SiGolSs* (except for *SiGolS5*) and 5 *SiGolSs* (except for *SiGolS2* and *SiGolS5*) showed the corresponding expression data under drought and waterlogging stresses, respectively. 13 *SiRSs* (except for *SiRS8* and *SiRS11*) showed the corresponding expression data under both drought and waterlogging stresses. Although some similar expression patterns were exhibited, *SiGolSs* and *SiRSs* showed complex expression patterns in response to drought and waterlogging stresses in two contrasting genotypes, as evidenced by the cluster analyses in the heatmaps (Figs 4 and 5).

Most of the *SiGolS* and *SiRS* genes showed similar expression pattern in response to drought stress between ZZM4782 (drought-sensitive, DS) and ZZM0635 (drought-tolerant, DT) varieties (Fig. 4). For instance, 3 *SiGolSs* (*SiGolS2*, *6* and *7*) and 7 *SiRSs* (*SiRS4*, *5, 6, 7, 13, 14* and *15*) were up-regulated, whereas 2 *SiGolSs* (*SiGolS1* and 4) and 4 SiRSs (*SiRS3*, *9, 10*, and *12*) were down-regulated, by drought stress in both genotypes. However, some genes showed different expression pattern between DS and DT varieties. *SiGolS3* was significantly induced at 3 d and 7 d after drought stress treatment in DT variety, while, it was slightly down-regulated under drought stress treatment in DS variety (Fig. 4).

Expression of most of the *SiGolS* and *SiRS* genes were repressed under waterlogging stress in both the waterlogging-tolerant (WT) variety Zhongzhi No. 13 and the waterlogging-susceptible (WS) variety ZZM0563 (Fig. 5). All the *SiGolSs* showed decreased transcripts under waterlogging stress at whole time points in the WT and WS varieties, except that *SiGols6* was instantaneously up-regulated at 3 h after waterlogging stress in the WT variety. Six *SiRSs* (*SiRS1*, *6, 7, 12, 13*, *and 14*) were down-regulated under waterlogging stress in both WT and WS varieties. *SiRS3* and 9 were up-regulated at 9 h and 15 h after waterlogging stress in WT and WS varieties. *SiRS2, 4* and *5* were down-regulated only at 3 h after waterlogging stress in WT and WS varieties. *SiRS10* exhibited relatively higher transcript accumulation at 3 h and 15 h after waterlogging stress in the WT variety, but showed no significant difference in the WS variety (Fig. 5).

**Figure 6:**
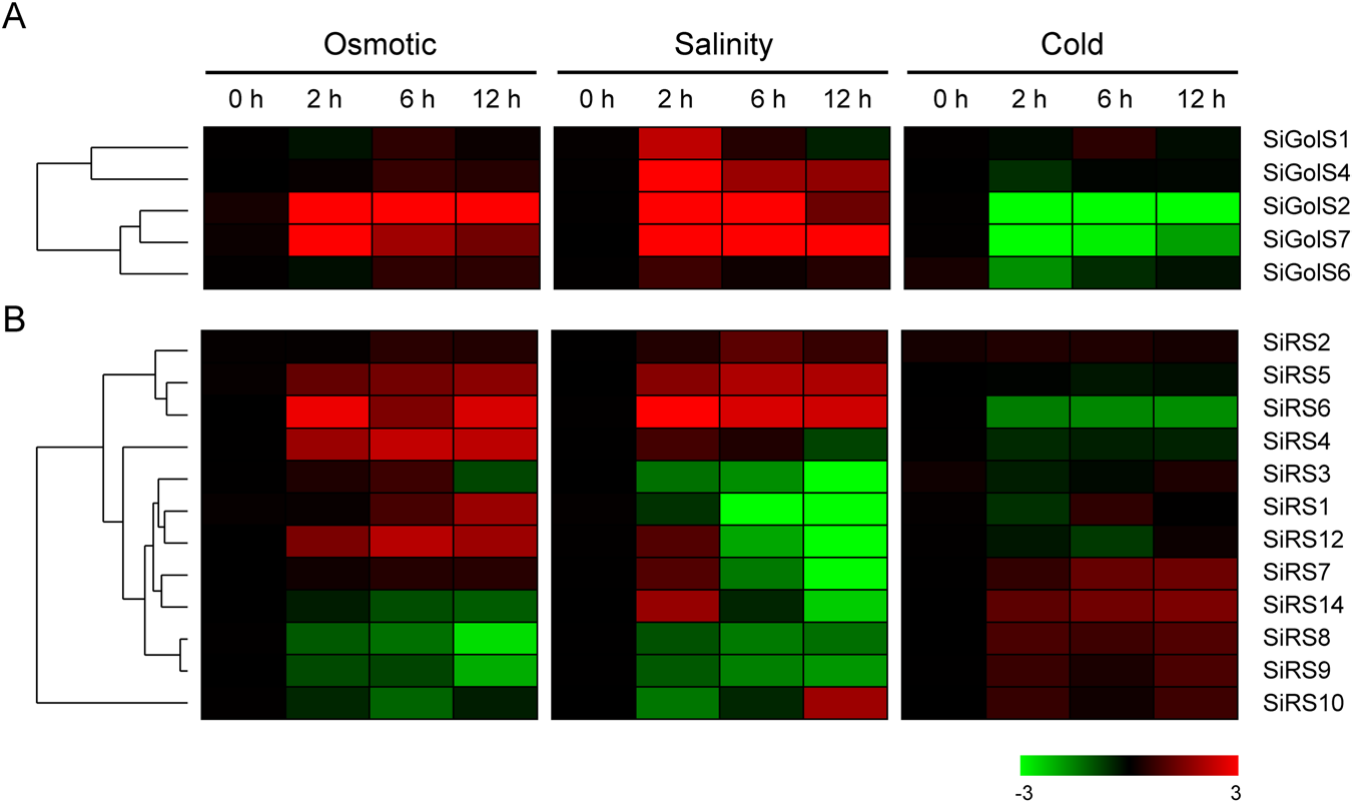
Expression profiles of *SiGolS* and *SiRS* genes under various abiotic stress treatments. Two-week-old seedlings were subjected to osmotic (15% PEG 6000), salt (150 mM NaCl), and cold (4 °C) stresses. The log2-transformed values (mean of three replicates) of the relative expression levels of the *SiGolS* (**A**) and *SiRS* (**B**) genes under abiotic stresses based on qPCR were used for creating the heatmap using Cluster 3.0. Changes in gene expression are shown in color as the scale. Original data was shown in Supplementary Figs S8 and S9.

To extend our understanding of *SiGolS* and *SiRS* genes in response to other important abiotic stresses impairing the sesame production, *5 SiGolSs* (*SiGolS1, 2, 4, 6, 7*) and 12 *SiRSs* (*SiRS1, 2, 3, 4, 5, 6, 7, 8, 9, 10, 12, 14*) that showed different expression patterns in different organs, and in response to abiotic stresses based on transcriptome data were chosen for further investigation of their expression patterns in shoot under osmotic, salinity, and cold treatments by qPCR. Under osmotic stress, *SiGolS2* and *SiGolS7* were significantly up-regulated (fold change > 2) during the whole treated time points, while the other 3 *SiGolSs* exhibited no significant change (Fig. 6A and Supplementary Fig. S8). Under salt treatment, 3 *SiGolSs* (*SiGolS2, 4*, and *7*) were significantly induced (fold change > 2) during the treatment period, whereas *SiGolS1* was only up-regulated at 2 h treatment. Under cold treatment, *SiGolS2* and *SiGolS7* were significantly repressed during the whole treated time points. As shown in Fig. 6B and Supplementary Fig. S9, 4 *SiRSs* (*SiRS4*, *5, 6*, and *12*) were significantly (fold change > 2) up-regulated, while *SiRS8* was significantly (fold change > 2) down-regulated during the entire osmotic stress time points. *SiRS5* and *SiRS6* were significantly induced (fold change > 2) during the salt treatment period, whereas *SiRS2, 7, 10, 12* and *14* were only up-regulated or down-regulated at particular time points. Under cold treatment, most of the *SiRSs* were not significantly affected, while *SiRS7* and *SiRS14* were significantly (fold change > 2) up-regulated. Together, these results indicated that most of the *SiGolS* and *SiRS* genes are active in response to osmotic and/or salt stresses, whereas slightly affected by cold stress (Fig. 6, Supplementary Figs S8 and S9).

### Galactinol and raffinose content in sesame exposed to osmotic stress

**Figure 7:**
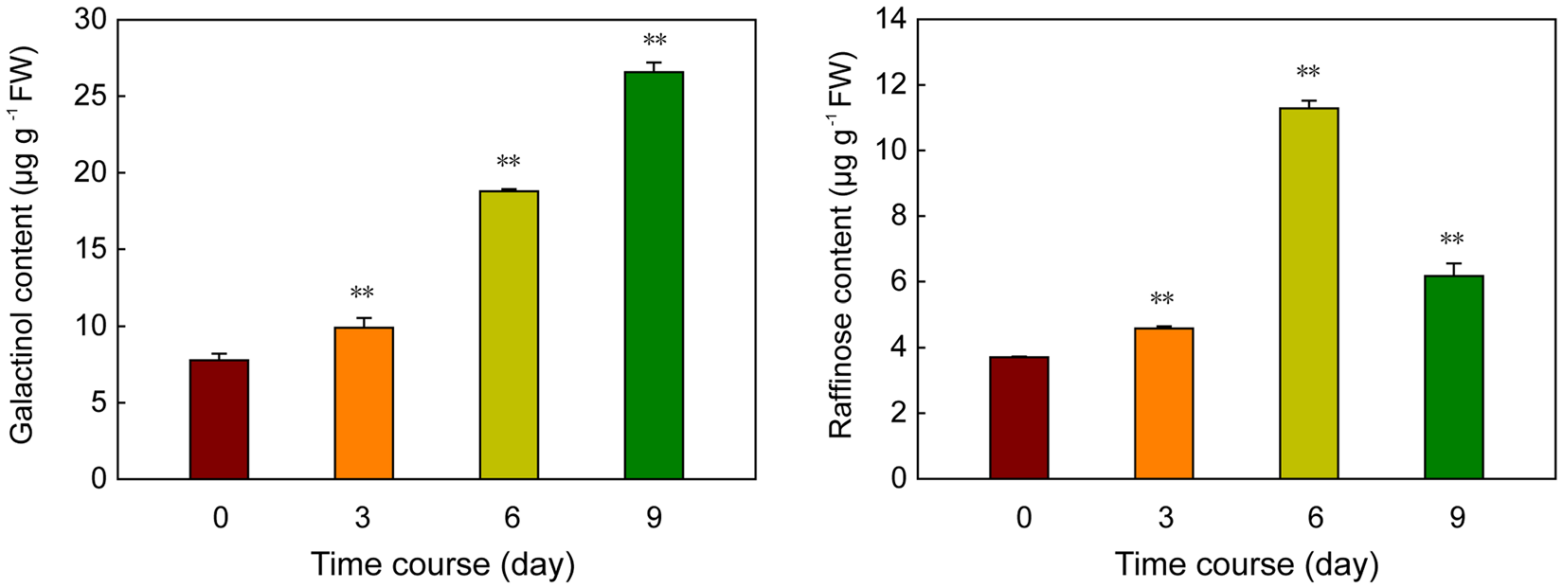
Changes in the content of galactinol and raffinose in sesame under osmotic stress. Two-week-old seedlings were subjected to osmotic (15% PEG 6000) stresses, and shoot samples were harvested at 0, 3, 6 and 9 days after treatment. Content of galactinol and raffinose was determined by LC-MS. Error bars indicate standard deviations based on three replicates. *P < 0.05; **P < 0.01, *t* test.

Finally, the changes in the content of galactinol and raffinose in sesame under osmotic stress treatment were investigated. Two-week-old seedlings were treated with 15% PEG 6000, and shoot samples were harvested at 0, 2, 5 and 9 days after treatment. As shown in Fig. 7, the content of both galactinol and raffinose clearly increased under osmotic stress, and the most obvious accumulation in galactinol and raffinose were observed at 6 days after treatment. During the osmotic stress treatment, the amounts of galactinol increased continuously. While the raffinose content peaked at 6 days and decreased subsequently.

## Discussion

Sesame is widely grown in arid and semi-arid areas facing frequent occurrences of drought and an increasing soil salinization due to intense use of irrigation and applied fertilizers^33^. Although sesame is a resilient crop that fairly resistant to several abiotic stresses including drought, salt, heat, it is highly sensitive to environmental stresses during its vegetative stage which directly affects its yield potential^27,28,34,35^. More importantly, the molecular mechanisms underlying sesame responses to abiotic stresses are poorly understood^36^. Raffinose family oligosaccharides (RFOs), which accumulate during seed development and plant exposed to abiotic stresses, perform a critical function in desiccation tolerance of developing seeds and plants^6,7^. Although some RFOs biosynthesis related genes (such as *GolS*s and *RSs*) have been studied in many plants^7,15,17,37^, less information is known about the GolS and RS gene families in sesame. Herein, a total of 7 SiGolSs and 15 SiRSs were genome-wide identified from sesame, which were classified into 5 and 6 subgroups, respectively, according to the phylogenetic relationship (Fig. 2). This classification is consistent with previous studies of GolS family in poplar, tomato and *Brachypodium distachyon*^21,38^. Furthermore, 13 RafS and 2 StaS (SiRS7 and 15) were further identified in SiRS gene family based on the existence of characteristic insertion of StaS^15^. The phylogenetic classification of GolS and RS was also supported by conserved motif and gene structure analyses. Protein and nucleotide sequence analyses showed that GolS and RS gene families harbored similar motifs and exon-intron organizations in the same subgroup (Supplementary Figs S3 and S6). These typical characteristics of these two gene families were also observed in other plants, such as maize, poplar, tomato and *Brachypodium distachyon*^20,21,38^. Collectively, similar conserved motifs and exon-intron organizations shared in the same subgroup indicate that SiGolSs and SiRSs in the same group had a closer relationship during the evolution process.

Based on transcriptome data, comprehensive expression profiles of *SiGolS* and *SiRS* genes at different developmental stages, or different tissues were revealed (Fig. 3). We found that some *SiGolS* and *SiRS* genes exhibited tissue- and developmental stage-specific expression patterns, indicating their possible roles in specific growth or developmental stages. For example, *SiRS8* and *10* exhibited specific higher expression in leaf and stem (Fig. 3B). RFOs accumulate during seeds development is thought to be important for desiccation tolerance during seed maturation and longevity in dehydrated state^18,39^. Seeds of the *AtRS4* and *AtRS5* double mutant showed a total loss of RFOs and five days delayed germination phenotype in darkness, suggesting that RFOs also act as a galactose store in seeds and are necessary for rapid germination in the dark^40^. Among sesame *GolS* and *RS* genes, 3 *SiGolSs* (*SiGolS3, 6* and *7*) and 2 *SiRSs* (*SiRS4* and *5*) displayed relatively higher transcripts during seed development, suggesting that these RFOs synthetic genes may be involved in the sesame seed development process.

RFOs were also found accumulated under multiple abiotic stress conditions and function as osmolytes to stabilise cell components, and/or act as reactive oxygen species (ROS) scavengers^3,9^. We also found galactinol and raffinose significantly accumulated under osmotic stress in sesame. Increasing evidence indicates that RFOs synthesis related genes, especially *GolS*, are important in the physiology of plant stress resistance. Expression analyses of *GolS* and *RS* gene family members in *Arabidopsis*, rice, maize, poplar, and tomato suggested that many *GolS* and *RS* genes showed transcriptional changes under drought, high-salinity, and cold stresses^17,19–21^. Moreover, transgenic plants analyses revealed special members of GolS and RS gene families as key players in plant abiotic stress resistance. *AtGolS2* was up-regulated by drought and salt stresses, overexpression of *AtGolS2* not only enhanced tolerance to drought, salt, chilling and oxidative stresses in transgenic *Arabidopsis*^7,17^, but also improved drought stress tolerance in the monocot model *Brachypodium distachyon* and rice^22,41^. Especially, overexpression of *AtGolS2* reduces yield losses under field drought conditions under different environmental conditions and in different rice genetic backgrounds, which suggests that AtGolS2 is a useful biotechnological tool to improve drought tolerance in rice^22^. Based on *in silico* analysis and our qPCR analysis, 6, 2, and 4 *SiGolSs* were regulated by drought stress (4 up-regulated genes and 2 down-regulated genes), osmotic stress (2 up-regulated genes), and salinity stress (4 up-regulated genes), respectively (Figs. 4 and 6). Among the 7 sesame *GolS* genes, *SiGolS2* and *SiGolS7* showed a closer phylogenetic relationship with *AtGolS2* (Fig. 2A), and exhibited amino acid identities of 81% and 70%, respectively, to the protein encoded by *AtGolS2*. Moreover, *SiGolS4* and *SiGolS7* were significantly up-regulated in both osmotic and salinity stresses (Fig. 6A and Supplementary Fig. S8), suggesting that these *SiGolS*s might be positively involved in drought and salt tolerances of sesame. Our study also found that 10, 8 and 11 *SiRSs* were regulated by drought stress (5 up-regulated genes and 5 down-regulated genes), osmotic stress (5 up-regulated genes and 3 down-regulated genes) and salt stress (4 up-regulated genes and 7 down-regulated genes), respectively (Figs 4 and 6). Among these genes, *SiRS5* and *SiRS*6 were commonly up-regulated by drought, osmotic, and salinity stresses. On the contrary, *SiRS8* and *SiRS9* were down-regulated in both osmotic and salinity stresses. Additionally, we found that 3 *SiGolSs* (*SiGolS2*, *6* and *7*) and 7 *SiRSs* (*SiRS4, 5, 6, 7, 13, 14* and *15*) could be induced by drought stress in different genotypes (Fig. 4). All these evidences demonstrated the implication of these genes in response to abiotic stresses in sesame, and therefore, could be further targeted for functional analysis. Interestingly, the expression of *SiGolS* and *SiRS* genes was slightly affected by cold stress except *SiGolS2* and *SiGolS7* (Fig. 6; Supplementary Figs S8 and S9), which could be explained by the fact that sesame was native to warm areas. Sesame is highly susceptible to waterlogging stress, and waterlogging is a significant environmental constraint to sesame production in China and Korea^32^. However, the expression of genes involved in RFOs biosynthesis under waterlogging stress is largely unknown. Herein, we provide the first insight into waterlogging-responsive of GolS and RS gene family members. Most of the *SiGolSs*, were down-regulated under waterlogging stress in two genotypes. GolSs act as a switch of inositol metabolism and RFO biosynthesis. Down-regulated of many *SiGolS* genes under waterlogging stress may divert *myo*-inositol away from the RFO synthetic pathway, thus participated in *O*-methyl-inositol (OMI) synthesis and act as a stress tolerance molecule^9^. These results presented here would be helpful for uncovering the function of RFOs synthetic pathway in abiotic stress resistance in sesame. In conclusion, 7 *SiGolS* and 15 *SiRS* genes from sesame have been characterized based on evolutionary, conserved protein motif, and gene structure analyses. The expression profiles of *SiGolS* and *SiRS* genes reveal their involvement in sesame seed development and responses to abiotic stresses. Together, these data will supply abundant information for functional characterization of *SiGolS* and *SiRS* genes and advance our understanding of RFOs-mediated abiotic stress tolerance in sesame.

## Methods

### Sequence identification and phylogenetic analysis

Protein sequences of genes involved in raffinose biosynthesis in *Arabidopsis*, such as *AtGolSs* and *AtRSs*, were used as queries to search against the protein database at Sinbase (*Sesamum indicum* genome database, http://ocri-genomics.org/Sinbase/index.html)^42^ by using BLASTP. Resulting sequences with an *E*-value of <1e−100 were analyzed manually in Pfam (http://pfam.sanger.ac.uk/) to validate the presence of Glyco_trans_8 Pfam (PF01501) for GolS proteins, or Raffinose_synthase Pfam (PF05691) for RafS and SatS proteins, respectively. *GolS* and *RS* proteins of representative sequenced plant species used in this study were obtained from the respective project databases (see below). *Arabidopsis thaliana*: The Arabidopsis Information Resource (TAIR), http://www.arabidopsis.org; *Oryza sativa subsp*. *japonica*: Rice Genome Annotation Project (RGAP), http://rice.plantbiology.msu.edu; *Brachypodium distachyon*, *Solanum lycopersicum*, *Populus trichocarpa*, and *Zea mays*: Phytozome, http://www.phytozome.net. The Neighbor-joining (NJ) phylogenetic trees were constructed with Clustalx 1.83 and MEGA5.05 software^43,44^.

### Gene structure and motif identification of SiGolSs and SiRSs

Exon and intron structures of these genes were investigated by comparing the coding sequences with their corresponding genomic sequences from Sinbase database, and visualized by using GSDS 2.0 (http://gsds.cbi.pku.edu.cn/index.php)^45^. The duplication pattern of each *SiGolS* and *SiRS* gene was analyzed using MCScanX software (http://chibba.pgml.uga.edu/mcscan2/) according to the previous description^46^. Conserved motifs in SiGolSs and SiRSs were identified using MEME v4.11.4 (http://meme-suite.org/tools/meme).

### Plant growth and stress treatment

To measure the transcript levels of the sesame GolS and RS family members under various abiotic stresses, seeds of sesame cultivar Zhongzhi No. 13 were germinated and grown hydroponically in a growth chamber with a 16 h light/8 h dark cycle. For osmotic and salt stress treatments, two-week old seedlings were treated with 15% PEG 6000 and 150 mM NaCl. For cold stress, seedlings were transferred to a growth chamber at 4 °C. Shoot samples from five randomly selected plants were collected (as one biological replicate) at 0 h (pretreatment), and at 2 h, 6 h and 12 h after stress treatments. For each treatment and time point, three replicates were used for RNA extraction.

### Expression profiles analyses of *SiGolSs* and *SiRSs*

Total RNA was isolated using the EASYspin Plus kit (Aidlab, China) according to the manufacturer’s instructions. For real-time quantitative RT-PCR (qPCR) analysis, first-strand cDNAs were synthesized from DNaseI-treated total RNA using the HiScript II 1st Strand cDNA Synthesis kit (Vazyme, China) according to the manufacturer’s instructions. Real-time quantitative RT-PCR was performed on Roche LightCycler 480 real-time PCR system using the ChamQ SYBR qPCR Master Mix (Vazyme, China) according to the manufacturer’s protocol. The sesame *Histone H3*.*3* gene (*SIN_1004293*) was used as the endogenous control^47^. The relative expression levels were calculated as described previously^48^. The qPCR assays were performed with three replicates. The gene-specific primers are listed in Supplementary Table S1.

Expression patterns of *SiGolS* and *SiRS* genes in capsule, leaf, root, stem, and seeds at different stages were examined in a set of transcriptome data downloaded from Sesame Functional Genomics Database (SesameFG, http://www.ncgr.ac.cn/SesameFG). Expression data of *SiGolS* and *SiRS* genes under drought stress were extracted from the transcriptome data of two sesame varieties (drought-tolerant cultivar ZZM0635 and drought-sensitive cultivar ZZM4782) under drought stress at flowering stage^31^. Expression data of *SiGolS* and *SiRS* genes under waterlogging stress were extracted from the transcriptome data of two sesame varieties (waterlogging-tolerant cultivar Zhongzhi No. 13 and the waterlogging-susceptible cultivar ZZM0563) under waterlogging stress at flowering stage^32^. The hierarchical cluster analyses of gene expression were performed using Cluster 3.0 software^49^, and heatmaps were visualized with TreeView^50^.

### Quantification of galactinol and raffinose content

Quantification of galactinol and raffinose content in sesame was performed by liquid chromatography-mass spectrometry (LC-MS) at Wuhan Metware Biotechnology Co.,Ltd (Wuhan, China) as described by^51^, with small modifications. Briefly, shoot samples from five plants were harvested after stress treatment and immediately frozen in liquid nitrogen. Then, samples were crushed and extracted overnight at 4 °C with 1.0 ml 70% aqueous methanol. After filtering, the extracts were analyzed by LC-MS. Details of the methods for the quantification of galactinol and raffinose content by LC-MS are provided in Supplementary Methods S1.

## Electronic supplementary material

Supplementary Information

Supplementary Dataset 1
