# Supplementary material for: Genome-wide identification and expression analyses of genes involved in raffinose accumulation in sesame
Source: Sci Rep. 2018 Mar 12;8:4331. doi: 10.1038/s41598-018-22585-2 (PMC5847563; doi:10.1038/s41598-018-22585-2)
Supplement: Supplementary file 1 — Supplementary Information [file 41598_2018_22585_MOESM1_ESM.pdf]

Genome-wide identification and expression analyses of genes involved in raffinose accumulation in sesame

Jun You[1], Yanyan Wang[1], Yujuan Zhang[1, 2], Komivi Dossa[1, 3], Donghua Li[1], Rong Zhou[1], Linhai Wang[1], Xiurong Zhang[1]*

1. Key Laboratory of Biology and Genetic Improvement of Oil Crops, Ministry of Agriculture, Oil Crops Research Institute, Chinese Academy of Agricultural Sciences, Wuhan, 430062, China

2. Special Economic Crop Research Center of Shandon Academy of Agricultural Sciences， Shandong Cotton Research Center, Jinan, 250100, China

3. Centre d'Etudes Régional pour l'Amélioration de l'Adaptation à la Sécheresse (CERAAS), BP 3320 Route de Khombole, Thiès, Sénégal

***Correspondence:**

Xiurong Zhang

E-mail: zhangxr@oilcrops.cn

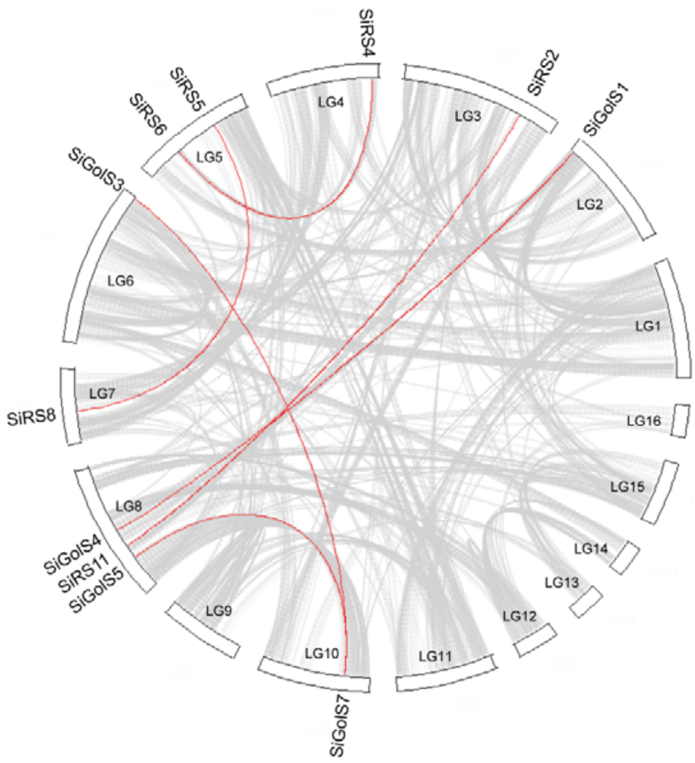

Supplementary Figure 1. Circle plot showing segmentally duplicated *SiGolS* and *SiRS* genes on sesame linkage groups. Grey lines indicated collinear blocks in whole sesame genome, and red lines indicated duplicated *SiGolS* or *SiRS* gene pairs.

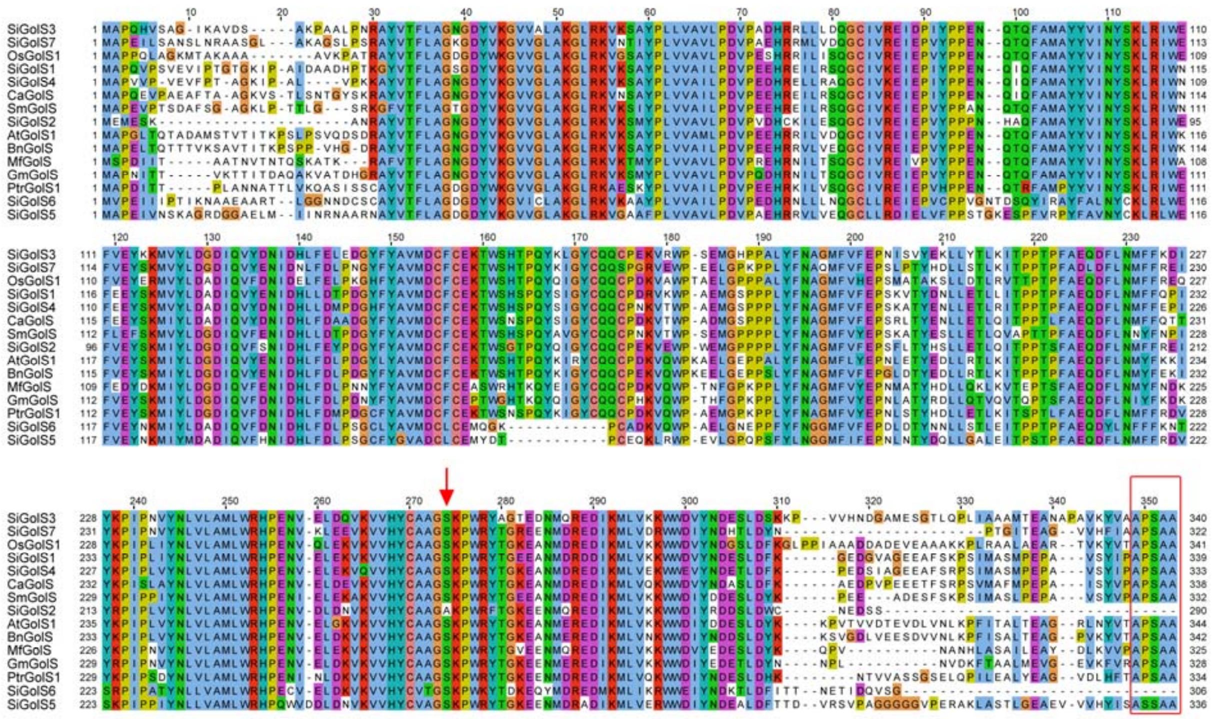

Supplementary Figure 2. The sequence alignment of SiGolSs and other reference GolS proteins. Multiple sequence alignment of GolS proteins from sesame (SiGolS1-7), *Arabidopsis* (AtGolS1, NP_182240.1), rice (OsGolS1, XP_015628833.1), *Populus trichocarpa* (PtGolS1, XP_002311774), *Coffea arabica* (CaGolS, ADM92588), *Salvia miltiorrhiza* (SmGolS, ACT34765), *Brassica napus* (BnGolS, ADG03603), *Medicago falcate* (MfGolS, ACM50915), and *Glycine max* (GmGolS, AAM96867). The putative serine phosphorylation site is shown by a red arrow. A characteristic hydrophobic pentapeptide (APSAA) is indicated by box.

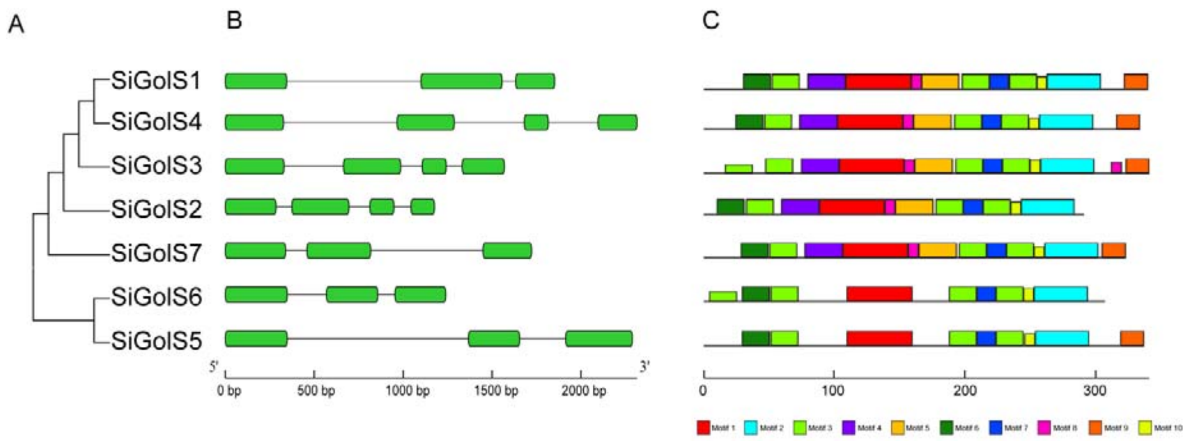

Supplementary Figure 3. Phylogenetic relationships, gene structure and motif compositions of SiGolSs. (A). The unrooted phylogenetic tree was created in MEGA5.05 software with the Neighbor-joining method with 1,000 bootstrap iterations based on full-length amino acids of SiGolSs. (B). Exon/intron structures of SiGolS genes. Exons and introns were represented by green boxes and black lines, respectively. Sizes of exons and introns could be estimated using the scale at bottom. (C). Schematic representation of the conserved motifs in the SiGolSs elucidated by MEME. Each motif was represented by a colored box numbered at the bottom. The black lines represented the non-conserved sequences. The length of protein could be estimated using the scale at the bottom. The details of individual motif were shown in Figure S4.

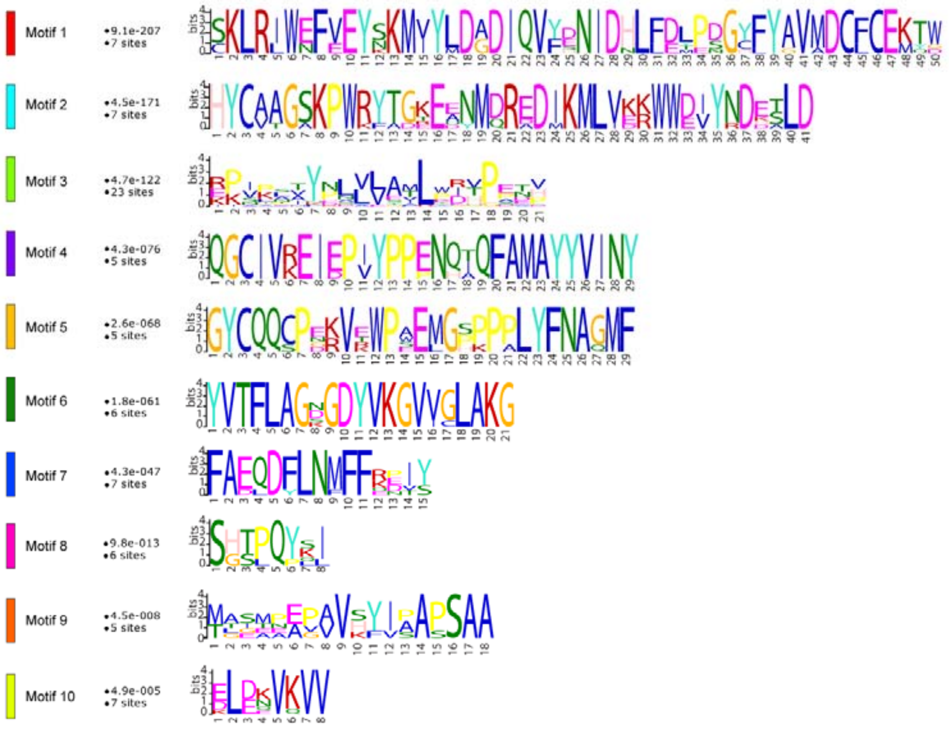

Supplementary Figure 4. Sequence logos for conserved motifs identified in SiGolSs by MEME analysis.

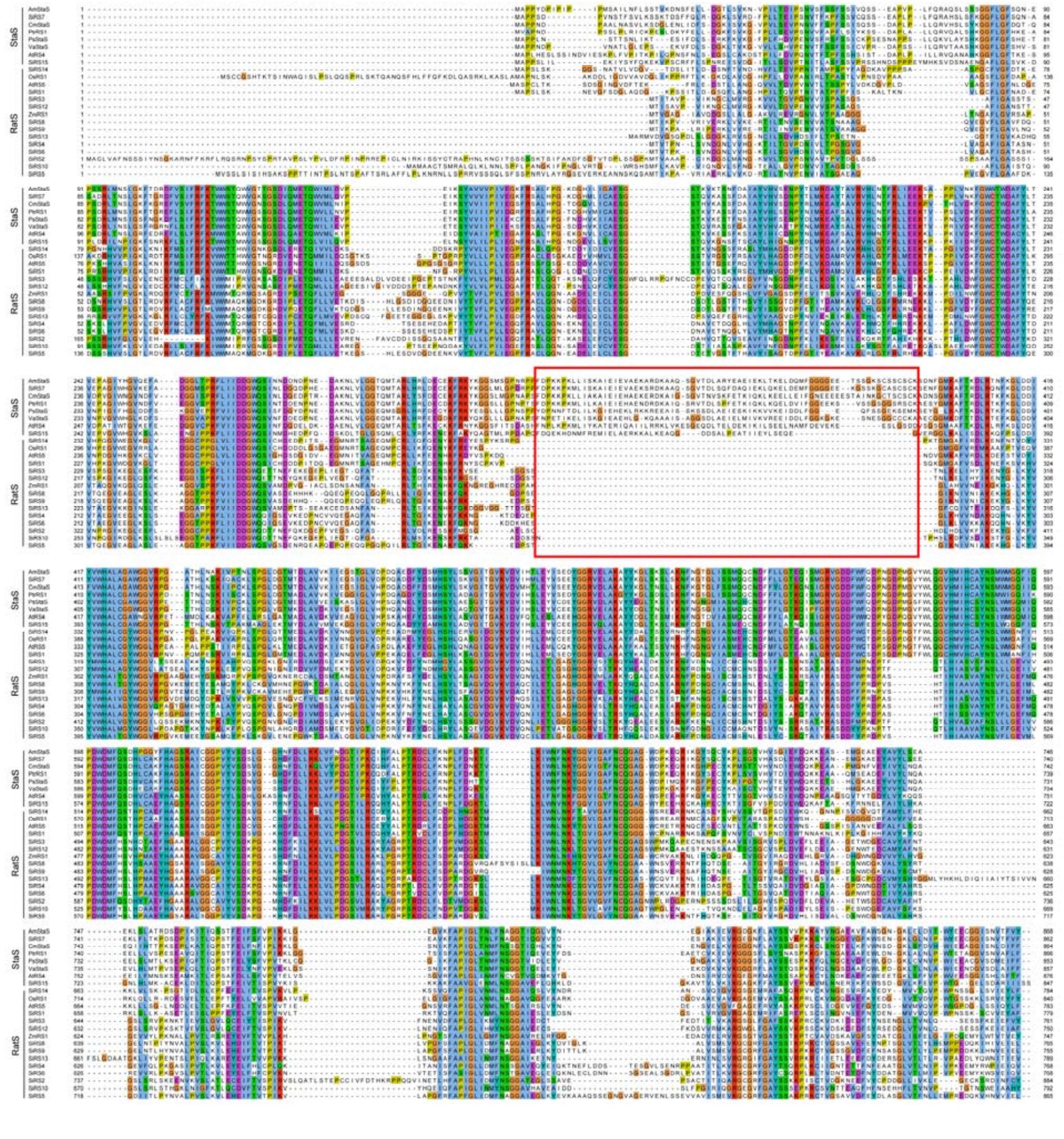

Supplementary Figure 5. The sequence alignment of SiRSs and other reference RS proteins. Multiple sequence alignment of RS proteins from sesame (SiRS1-15), *Arabidopsis* (AtRS4, NP_192106; AtRS5, NP_198855), *Populus trichocarpa* (PtrRS1, XP_006386712), rice (OsRS1, XP_015621501), maize (ZmRS1, NP_001105775), *Vigna angularis* (VaStaS, CAB64363), *Pisum sativum* (PsStaS, CAC38094), *Cucumis melo* (CmStaS, XP_008451468) and *Alonsoa meridionalis* (AmStaS, CAD31704). The characteristic insertion of StaS is indicated by box.

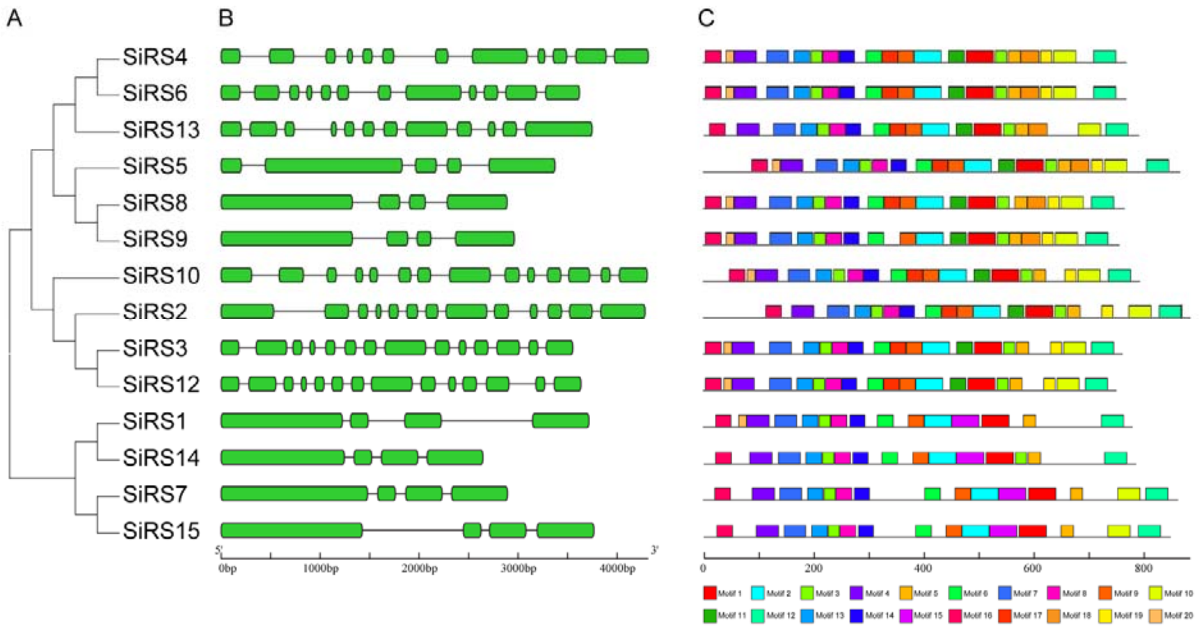

Supplementary Figure 6. Phylogenetic relationships, gene structure and motif compositions of SiRSs. (A). The unrooted phylogenetic tree was created in MEGA5.05 software with the Neighbor-joining method with 1,000 bootstrap iterations based on full-length amino acids of SiRSs. (B). Exon/intron structures of SiRS genes. Exons and introns were represented by green boxes and black lines, respectively. Sizes of exons and introns could be estimated using the scale at bottom. (C). Schematic representation of the conserved motifs in the SiRSs elucidated by MEME. Each motif was represented by a colored box numbered at the bottom. The black lines represented the non-conserved sequences. The length of protein could be estimated using the scale at the bottom. The details of individual motif were shown in Figure S7.

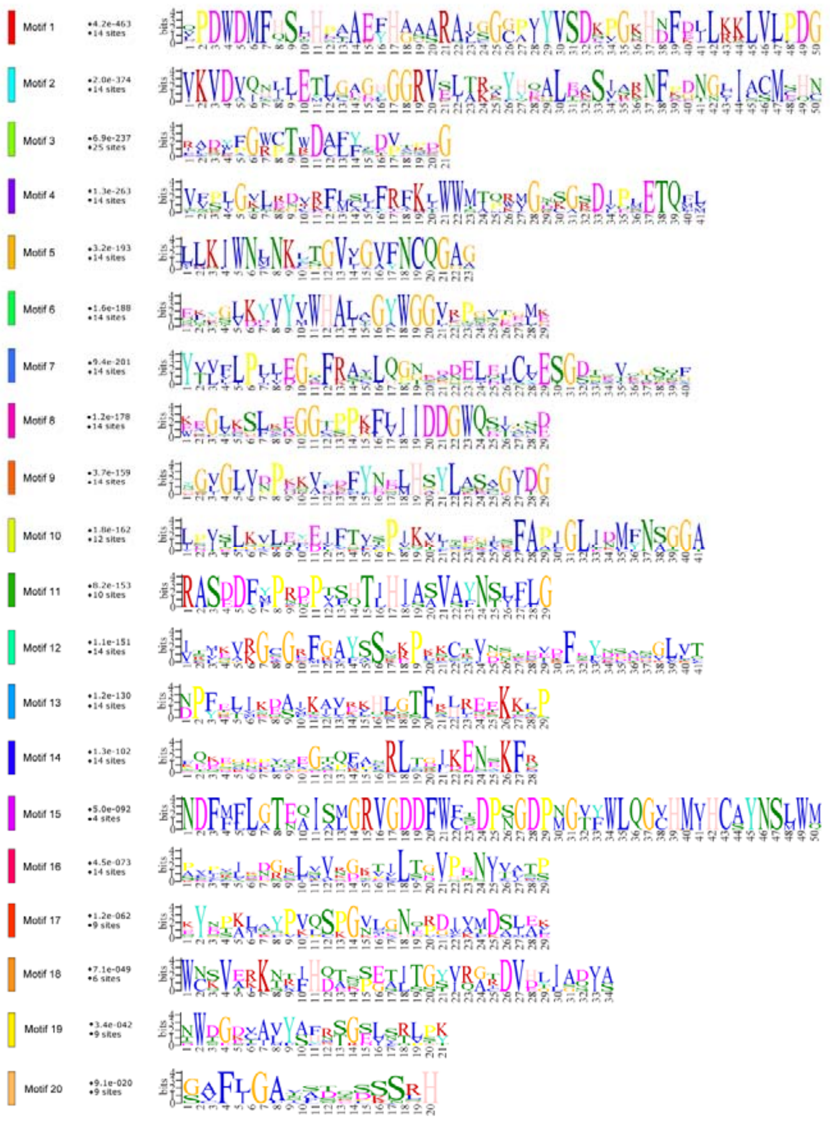

Supplementary Figure 7. Sequence logos for conserved motifs identified in SiRSs by MEME analysis.

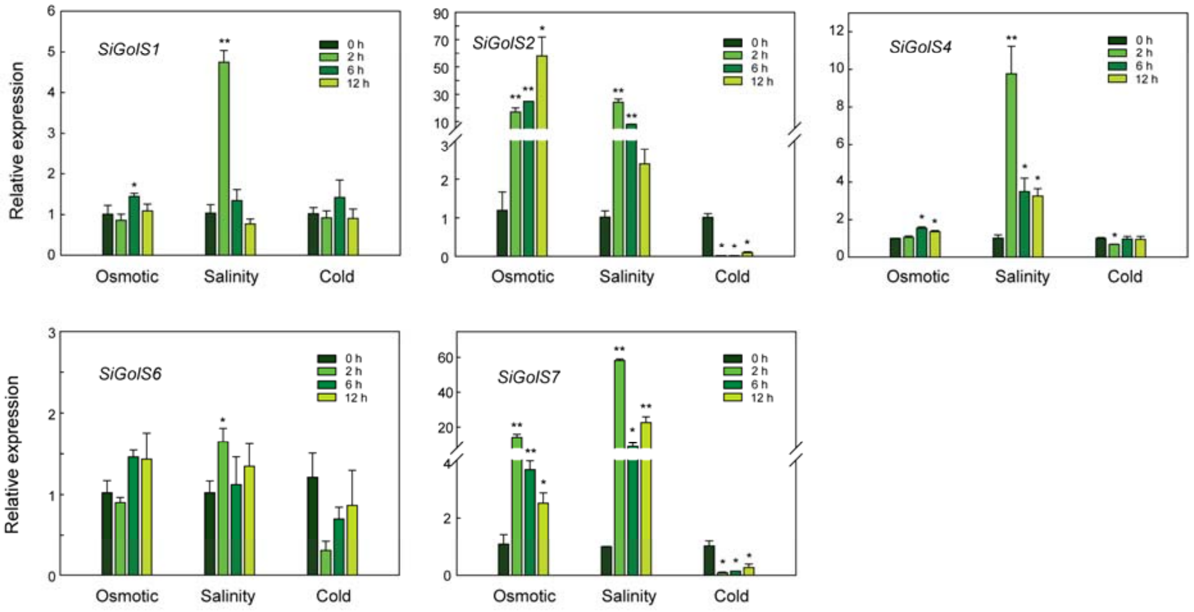

Supplementary Figure 8. Expression profiles of *SiGolS* genes under osmotic, salt and cold stresses. Two-week-old seedlings were subjected to osmotic (15 % PEG 6000), salt (150 mM NaCl), and cold (4ºC) stresses. Relative expression levels of *SiGolS* genes were analyzed by quantitative real-time RT-PCR (qPCR), using sesame *SiH3.3* gene as the internal control. Error bars indicate standard deviations (SD) based on three replicates. *$P < 0.05$; **$P < 0.01$, *t* test.

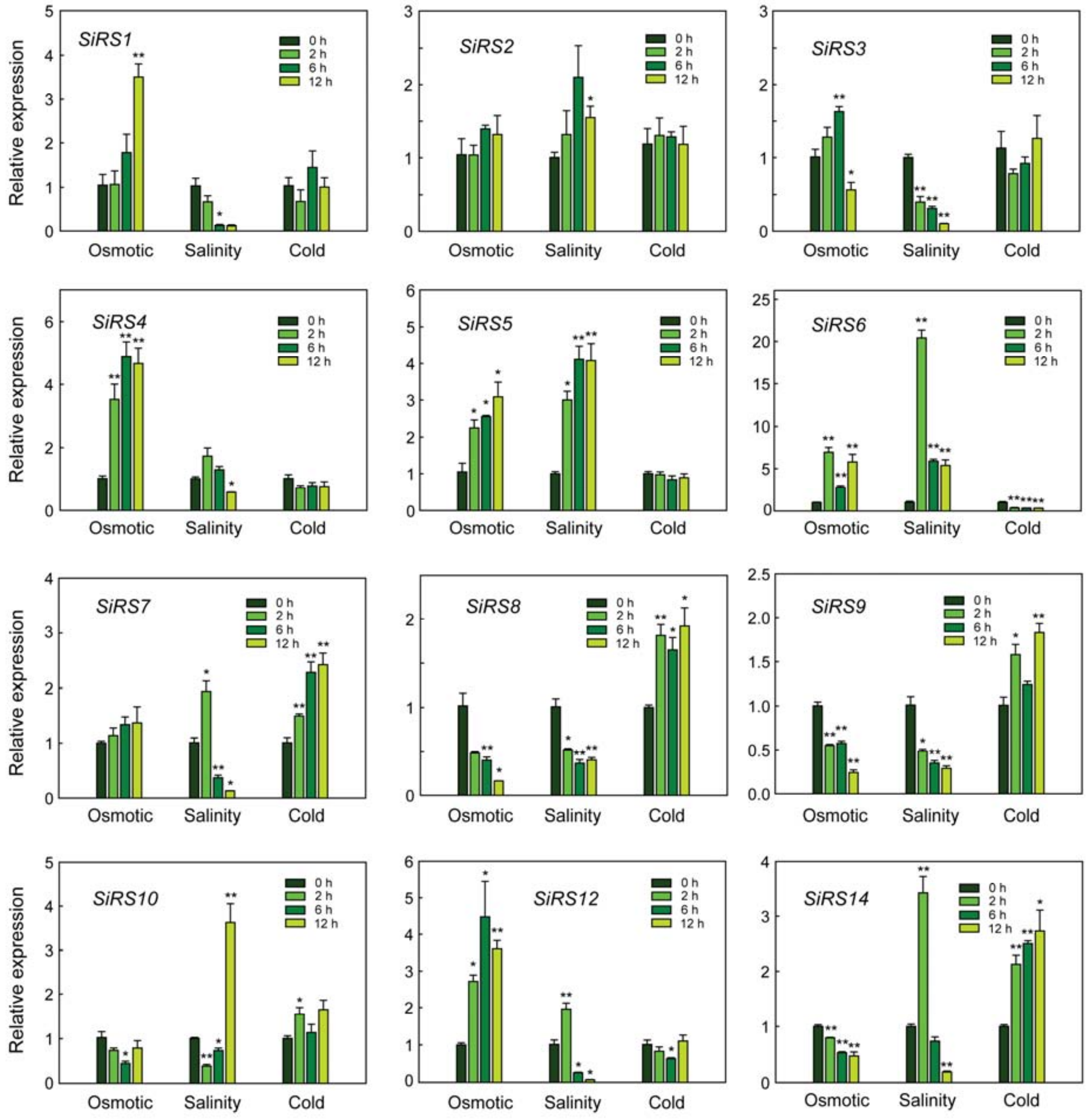

Supplementary Figure 9. Expression profiles of *SiRS* genes under osmotic, salt and cold stresses. Two-week-old seedlings were subjected to osmotic (15 % PEG 6000), salt (150 mM NaCl), and cold (4ºC) stresses. Relative expression levels of *SiRS* genes were analyzed by quantitative real-time RT-PCR (qPCR), using sesame *SiH3.3* gene as the internal control. Error bars indicate standard deviations (SD) based on three replicates. **P* < 0.05; ***P* < 0.01, *t* test.

Supplementary Method

Quantification of galactinol and raffinose content

1. Chemicals

Gradient grades of methanol, acetonitrile, and acetic acid were purchased from Merck Company, Germany (www.merckchemicals.com). Raffinose (CAS: 512-69-6) and galactinol (CAS: 3687-64-7) were purchased from Sigma-Aldrich, USA (www.sigmaaldrich.com/united-states.html).

2. Standard Solution and Calibration Curve

Stock solutions of raffinose and galactinol were individually prepared by dissolving each standard substance in 70% methanol to give a final concentration of 1000 ppm. Working standard solutions were prepared by stepwise dilution of this standard solution with 70% methanol in water, to produce final concentrations for each analysis in the range of 500 ppb to 0.5 ppb.

3. Sample preparation and extraction

The freezed sample was crushed using a mixer mill (MM 400, Retsch) with a zirconia bead for 1.5 min at 30 Hz. 100 mg powder was weighted and extracted overnight at 4°C with 1.0 ml 70% aqueous methanol. Following centrifugation at 10, 000 g for 10 min, the extracts were absorbed (CNWBOND Carbon-GCB SPE Cartridge, 250 mg, 3 mL; ANPEL, Shanghai,China, www.anpel.com.cn/cnw) and filtrated (SCAA-104, 0.22 μm pore size; ANPEL,Shanghai, China, http://www.anpel.com.cn/) before LC-MS analysis.

4. HPLC Conditions

The sample extracts were analyzed using an LC-ESI-MS/MS system (HPLC, Shim-pack UFLC SHIMADZU CBM30A system, www.shimadzu.com.cn; MS, Applied Biosystems 4500 Q TRAP, www.appliedbiosystems.com.cn/). The analytical conditions were as follows, HPLC: column, Waters ACQUITY UPLC HSS T3 C18 (1.8 μm, 2.1 mm*100 mm); solvent system, water (0.04% acetic acid): cetonitrile (0.04% acetic acid); gradient program, 100:0 (V/V) at 0 min, 5:95 (V/V) at 11.0 min, 5:95(V/V) at 12.0 min, 95:5(V/V) at 12.1 min, 95:5(V/V) at 15.0 min; flow rate, 0.40 mL/min; temperature, 40°C; injection volume: 5 μl. The effluent was alternatively connected to an ESI triple quadrupole linear ion trap (Q TRAP)-MS.

5. ESI-Q TRAP-MS/MS

LIT and triple quadrupole (QQQ) scans were acquired on a triple quadrupole linear ion trap mass spectrometer (Q TRAP), API 4500 Q TRAP LC/MS/MS System, equipped with an ESI Turbo Ion-Spray interface, operating in a positive ion mode and controlled by Analyst 1.6 software (AB Sciex). The ESI source operation parameters were as follows: ion source, turbo spray; source temperature 550°C; ion spray voltage (IS) 5500 V; ion source gas I (GSI), gas II(GSII), curtain gas (CUR) were set at 55, 60, and 25.0 psi, respectively; the collision gas(CAD) was high. Instrument tuning and mass calibration were performed with 10 and 100 μmol/L polypropylene glycol solutions in QQQ and LIT modes, respectively. QQQ scans were acquired as MRM experiments with collision gas (nitrogen) set to 5 psi. DP and CE for individual MRM transitions were done with further DP and CE optimization. The MRM transitions of raffinose was m/z 503.4 (Q1)/179.1 (Q3), the DP and CE was -80 and -30 respectively. The MRM transitions of galactinol was m/z 341.1 (Q1)/179.1 (Q3), the DP and CE was -80 and -20 respectively. A specific set of MRM transitions were monitored for each period according to the metabolites eluted within this period.
